# The influence of methylphenidate on the power spectrum of ADHD children – an MEG study

**DOI:** 10.1186/1471-244X-5-29

**Published:** 2005-07-26

**Authors:** Christian Wienbruch, Isabella Paul, Susanne Bauer, Hermann Kivelitz

**Affiliations:** 1 Clinical Psychology, University of Konstanz, Konstanz, Germany; 2 Pratice for Child and Adolescent Psychiatry and Psychotherapy, Ettlingen, Germany

## Abstract

**Background:**

The present study was dedicated to investigate the influence of Methylphenidate (MPH) on cortical processing of children who were diagnosed with different subtypes of Attention Deficit Hyperactivity Disorder (ADHD). As all of the previous studies investigating power differences in different frequency bands have been using EEG, mostly with a relatively small number of electrodes our aim was to obtain new aspects using high density magnetoencephalography (MEG).

**Methods:**

35 children (6 female, 29 male) participated in this study. Mean age was 11.7 years (± 1.92 years). 17 children were diagnosed of having an Attention-Deficit/Hyperactivity Disorder of the combined type (ADHDcom, DSM IV code 314.01); the other 18 were diagnosed for ADHD of the predominantly inattentive type (ADHDin, DSM IV code 314.0). We measured the MEG during a 5 minute resting period with a 148-channel magnetometer system (MAGNES™ 2500 WH, 4D Neuroimaging, San Diego, USA). Power values were averaged for 5 bands: Delta (D, 1.5–3.5 Hz), Theta (T, 3.5–7.5 Hz), Alpha (A, 7.5–12.5 Hz), Beta (B, 12.5–25 Hz) and Global (GL, 1.5–25 Hz).). Additionally, attention was measured behaviourally using the D2 test of attention with and without medication.

**Results:**

The global power of the frequency band from 1.5 to 25 Hz increased with MPH. Relative Theta was found to be higher in the left hemisphere after administration of MPH than before. A positive correlation was found between D2 test improvement and MPH-induced power changes in the Theta band over the left frontal region. A linear regression was computed and confirmed that the larger the improvement in D2 test performance, the larger the increase in Theta after MPH application.

**Conclusion:**

Main effects induced by medication were found in frontal regions. Theta band activity increased over the left hemisphere after MPH application. This finding contradicts EEG results of several groups who found lower levels of Theta power after MPH application. As relative Theta correlates with D2 test improvement we conclude that MEG provide complementary and therefore important new insights to ADHD.

## Background

Attention Deficit Hyperactivity Disorder (ADHD) is characterized by difficulties concentrating, completing assigned tasks, keeping track of things, waiting one's turn or sitting still. Three subtypes are classified in the DSM IV [1] ADHD of the predominantly inattentive type, ADHD of the predominantly hyperactive type and a combined type. The prevalence of ADHD is estimated to lie between 3 and 5% of all school children with a stronger tendency for boys to be diagnosed [1] However, Scahill and Schwab-Stone [2] investigated data from 13 studies and found prevalence to vary between 2 and 14.9%, depending on diagnostic tools and community sample. An increase in prevalence has been observed throughout the last years, which might be related to a change in diagnostic criteria and the introduction of ADHD predominantly hyperactive type in the DSM IV. All ADHD subtypes are generally treated the same way, the prescription of Methylphenidate (MPH) [3]. MPH has shown to be effective in 75–90% of ADHD children [4]. In line with increasing prevalence estimates, the usage of MPH has increased several fold during the last years in the USA [5] as well as in Germany [6].

It has been stated by several authors, that ADHD is related to cortical hypoarousal [7-10]. The mechanism behind this possible hypoarousal is not yet clarified. However, evidence from SPECT studies [11,12] and the mere fact that MPH – a psychostimulant – is an effective treatment of ADHD symptoms suggest there is a deficit in the dopaminergic neurotransmitter system. As SPECT studies (e.g. [13]) have shown, ADHD patients seem to have a higher number of DAT receptors, which are responsible for dopamine re-uptake, and in consequence have less dopamine available in the synaptic gap. MPH is a potent blocker of DAT receptors.

It has previously been shown that some children respond more to MPH than others, or that children with different sub-diagnoses react different. Clarke and colleagues [14,15] compared EEG power in different frequency bands of good and poor responders to MPH and found a cortical activation profile suggesting that good responders are more cortically hypoaroused than poor responders. The authors assume that MPH is most effective for children who are cortically hypoaroused. Loo and co-workers [16] conclude from their results that there are different electrophysiological correlates to MPH for good responders and poor responders. They also compared EEG power in different frequency bands and found that reponders showed a decrease in Theta and Alpha activity, as well as an increase in Beta activity, while poor responders showed the opposite pattern. Clarke and colleagues [17-19] investigated cortical differences between ADHD children of the combined type (ADHDcom) and the predominantly inattentive type (ADHDin). Generally, they found ADHDcom children having higher slow wave activity than ADHDin children. The authors concluded from their results that children with ADHDcom are more cortically hypoaroused than children with ADHDin. They hypothesized that ADHDcom might be related to frontal lobe dysfunctions, while children with ADHDin may have other forms of CNS functioning.

Due to the findings that MPH is more effective for some children than for others and since the cortical profiles of ADHD subtypes seem to differ, it is advisable to study the effects of MPH on cortical processing more closely. One desirable outcome of these studies might be to identify cortical indicators in order to differentiate between children who are good responders and those who are poor responders *before *they are treated with amphetamines. Another value lies in elucidating etiological factors of ADHD. Differential effects for the different subtypes can help understanding the underlying cause of the disorder.

The present study was dedicated to investigate the influence of MPH on cortical processing. All of the previous studies investigating power differences in different frequency bands have been using EEG, mostly with a relatively small number of electrodes. Our aim was to obtain new aspects using high density magnetoencephalography (MEG). MEG comprises several advantages over EEG. First of all, the magnetic fields measured are not as biased by low skull conductivity as electrical potentials. Second, MEG is reference-free. Unless EEG analysis is done using average reference (which only is reliable if the recording of the reference electrode is flawless), there will always be an influence on cortical effects produced by the reference type chosen. Third, MEG using magnetometers by definition mostly reflects cortical activity. Subcortical activity is often too weak to be detected. Thus, the complexity of the detected signals is reduced. Fourth, MEG mainly reflects cortical activity from structures, which have a tangential orientation to the surface of the head. Thus, the activity measured most likely stems from circumscribed cortical structures in the walls of the gyri and sulci, whereas potential differences measured by EEG can originate from both radial and tangential oriented fibers from the whole brain. Thus it is rather likely that MEG shows complementary but similar information than EEG.

## Methods

### Subjects

35 children (6 female, 29 male) participated in this study. Mean age was 11.7 years (± 1.92 years). 17 children were diagnosed of having an Attention-Deficit/Hyperactivity Disorder of the combined type (ADHDcom, DSM IV code 314.01); the other 18 were diagnosed for ADHD of the predominantly inattentive type (ADHDin, DSM IV code 314.0). Diagnoses were made by a paediatrician specialized in child psychiatry. All children and parents gave their written informed consent to participate according to the World Medical Association Declaration of Helsinki – Ethical Principles for Medical Research Involving Human Subjects [20].

### Procedure

We measured the effect of Methylphenidate (MPH) on the MEG during a 5 minute resting period (subjects being relaxed but awake). The behavioural performance with and without medication was measured by a highly demanding attention test (D2 test of attention, [21]). Dosage of methylphenidate was based on the body weight of the child (0.1–0.5 mg/kg/day). To ensure that medication and not the mere administration of a pill had an effect, we chose a placebo design. Placebo and methylphenidate were applied by a pediatrician in form of pills that looked identical (placebo dosage was matched with MPH dosage).

**Figure 1:**
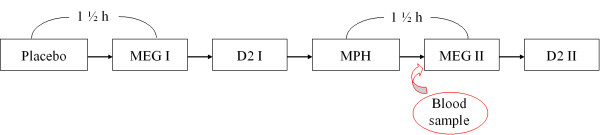
Study design.

The overall-design was the following (Fig. 1): due to feasibility it was decided to run the whole procedure within one day. Therefore it was not possible to counterbalance the application time of placebo and MPH, since MPH takes several days to be untraceable in the blood. In order to have an objective measure of the concentration of MPH in the blood serum, blood samples were taken from the children an hour after drug administration. The blood serum was separated right after being taken and was then deep frozen. MPH serum concentrations were measured in an external professional laboratory.

### MEG recording

Recording was done with a 148-channel magnetometer (MAGNES™ 2500 WH, 4D Neuroimaging, San Diego, USA). A subject specific headframe coordinate system was defined by means of 3 anatomical landmarks also called 'head fiducials' (left and right preauricular and nasion). These head fiducials, five coils and the subject's head shape were digitized with a Polhemus 3Space^® ^Fasttrack prior to each measurement. The subject's head position relative to the pickup coils of the sensor was estimated before and after each measurement to ensure that no large movements occurred during the measurement. As the position of the pickup coils are known in device coordinates, this procedure also allows their transformation into the headframe coordinate system.

The children were lying supine in a comfortable position in a magnetically shielded room (Vakuumschmelze Hanau). They were instructed to lie still for 5 minutes and to fixate a point at the ceiling in order to keep eye movements minimal. Continuous data sets were recorded with a real high-pass filter of 0.1 Hz and a sampling rate of 678.17 Hz (bandwidth 200 Hz). Real time noise reduction procedures, i.e. the subtraction of the signal measured by 8 reference channels, multiplied with sets of weight factors for each of the 148 magnetometers, were applied during acquisition before and directly after analog/digital (AD) conversion. This noise reduction procedure affects signals of interest originating in the brain or the body much less than the presence of a cancellation coil in a standard gradiometer detector.

For artifact control, eye movements (EOG) were recorded from four electrodes attached to the left and right outer canthus and above and below the right eye. A Synamps amplifier (NEUROSCAN™) served for the recording of the EOG. A video camera installed inside the chamber allowed monitoring the children's behaviour and compliance at any time throughout the experiment.

### D2 test

Immediately after the MEG recording, each child performed the D2 test in a quiet room. The test involves finding and marking the letter "d" within a string of letters ("d" and "p"), only when *2 *dashes are arranged either individually or in pairs above and below "d". A high amount of attention is necessary to perform the task successfully, since not only the letter "d" is orthographically similar to the letter "p", but because there are many distractor letters "d" with more than 2 dashes. Additionally, a time limit is set for finding as many D2s with as little errors as possible.

### Data analysis MEG

Global noise was filtered offline from the MEG data by subtracting external, non-biological noise recorded by 11 MEG reference channels. Before subtraction, reference channels were multiplied with individually calculated fixed weight factors. Again, noise reduction procedures have no or little influence on the biological signal because the distance of the reference set to the subjects head is relatively large (mean = 25.8 cm, std = 6.00 cm, min = 15.5 cm, max = 36.5 cm) compared to the distance between sensors and head, which is usually much smaller. The data was then split into epochs of 2500 ms length and was corrected for eye and cardiac artefacts by subtracting the moving average cardiac and vertical EOG signal from the data. All epochs with an MEG level > 3.5 pT between the minimum and maximum on one or more MEG channels after artefact correction were rejected. A fast fourier transformation (FFT) was computed for all epochs.

**Figure 2:**
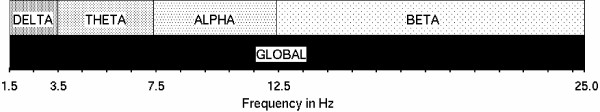
Power band definition.

For each subject the average power was calculated across channels for 6 cortical regions (frontal, temporal and occipital; left and right, respectively). In EEG, electrode positions are comparable across subjects, since they are usually defined in relation to fixed anatomical landmarks on the head. This is not true for MEG sensor coils. Therefore averages of MEG measures of the same coil across subjects add additional variance. This variance is closely related to the variance of sensor coil positions between subjects. In order to ensure that the same cortical regions were covered, subject specific channel-groups were selected. We defined 6 landmarks in the headframe-based coordinate system. In the second step, we determined 6 corresponding channels for each subject and each measurement that were closest (smallest Euklidean distance) to the previously defined landmarks. These 6 channels served as centre channels of our subject specific channel groups, consisting of either 15 (occipital) or 20 (frontal, temporal) channels. Channels were selected by being nearest neighbours to the centre channel of the respective channel group. Within these channel-groups the power values were averaged for 5 bands (see Fig. 2) and normalized to the size of the frequency bin: Delta (D, 1.5–3.5 Hz), Theta (T, 3.5–7.5 Hz), Alpha (A, 7.5–12.5 Hz), Beta (B, 12.5–25 Hz) and Global (GL, 1.5–25 Hz). The power values of the Delta, Theta, Alpha, and Beta frequency bands were normalized to the global power yielding relative power values. Additionally, T/A and T/B ratios were calculated.

### Data analysis D2 test

The total number of correctly marked items was used to determine the individual child's attention level. Raw values were expressed in percentiles (derived from age-matched norm samples), in order to achieve age-independent test scores. Improvement of attention was determined by subtracting the test score after placebo application from the test score after MPH application. Further, the subjects were divided into good responders (ADHDg) and poor responders (ADHDp): a child was classified as ADHDg, if the improvement was larger than 30 percentiles (this was slightly more than 1 standard deviation).

### Statistical analysis

To see if D2 test performance improved after medication, a one-way repeated measure ANOVA (analysis of variance) was calculated. D2 test score was dependent variable, TIME (pre, post) was repeated measure.

To quantify the influence of medication on the power bands in the different cortical regions, a mixed model analysis was computed using the statistical package SAS^®^9. Covariance parameters were estimated with the restricted maximum likelihood method (REML). Relative power values (D, T, A, B, T/A, T/B) as well as the global power (GL), were defined as dependent variables. TIME (pre, post), HEMISPHERE (left, right), REGION (fontal, temporal, occipital) and either DSMtype (combined, inattentive) or Response (responders, non-responders) were fixed effects. Depending on which fixed effect was used in the analysis, the factor *Patient *either nested in *DSMtype *or *Response *was used as random factor. Variance structure was *variance components *(VC). Post-hoc testing was performed following Tukey-Kramer. In cases of non-significant post-hoc tests, uncorrected p-values are reported.

Finally, all power values (all bands and all regions) after placebo were subtracted from the respective power values after MPH application. This gave us a measure of MPH induced power changes. Correlations were calculated between the power changes, MPH blood serum concentration, age in months and D2 test improvement.

*Only significant main effects, interactions and post-hoc tests are reported*. For the mixed model analysis, *only significant interactions with D2-response or DSM-type are reported*, since the aim was to reveal cortical differences between the different DSM-subtypes and children, who respond well to MPH compared to those who do not profit as much. All plots show standard errors.

## Results

### D2-test

A main effect for TIME was found (F(1,34) = 86.87, p < 0.001). D2-test performance was significantly higher after the application of MPH (73.1 percentiles) than before MPH (41.2 percentiles).

**Figure 3:**
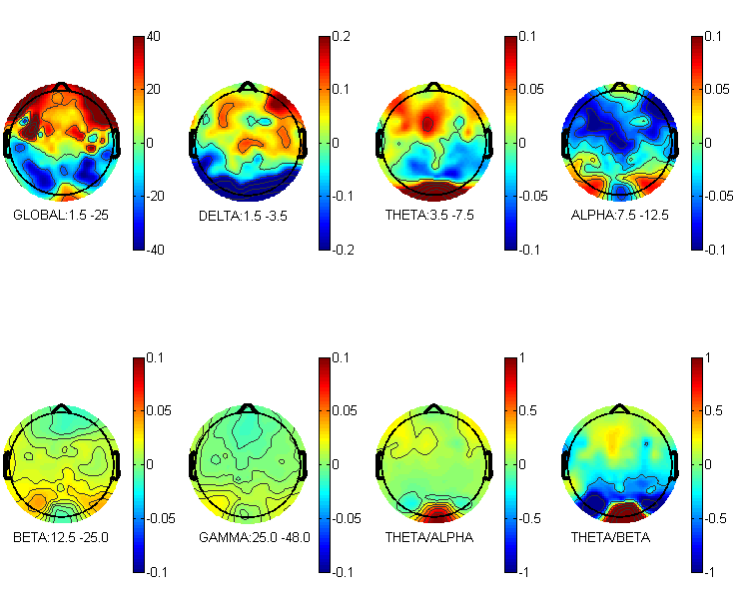
**Difference of global power – MPH minus Placebo. **The difference of the grand average global and relative power (delta, theta, alpha, beta, and gamma) between the MPH condition and the placebo condition calculated over the 35 subjects. It can clearly be seen that the global power is strongest over frontal regions (global power is given in ft/vHz. Delta, Theta, Alpha, Beta and Gamma are relative measures, normalized to the Global power. Theta/Alpha and Theta/Beta are ratios of the relative power, which is identical to ratios of the power).

**Figure 4:**
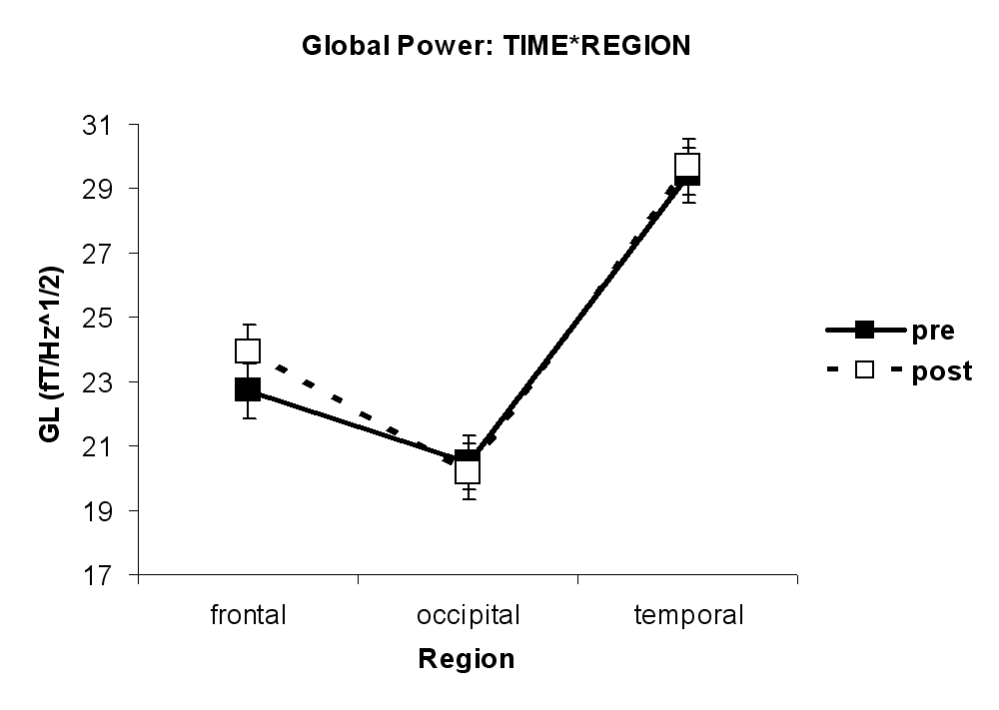
Interaction TIME*REGION for global power. MPH effects were only found in frontal regions (p = 0.05) with higher amplitudes after MPH than before MPH. No differences were observed in temporal or occipital regions.

In order to investigate, which cortical region would be most affected by MPH application, an analysis over all ADHD children was performed, no matter what subtype they were diagnosed. The analysis yielded the significant interaction TIME*REGION (F(1,33) = 4.46, p = 0.015) for the dependent variable Global Power. As can be seen in figures 3 and 4, MPH effects were only found in frontal regions (p = 0.05) with higher amplitudes after MPH than before MPH. No differences were observed in temporal or occipital regions. Thus, further analysis was restricted to frontal channel groups.

### Results frontal channels

#### Global power

The main effect TIME (F(1,33) = 7.53, p = 0.0098) was found. Global power amplitude was higher after MPH (23.9 ft/Hz^/2) than before MPH (22.7 ft/Hz^/2).

**Figure 5:**
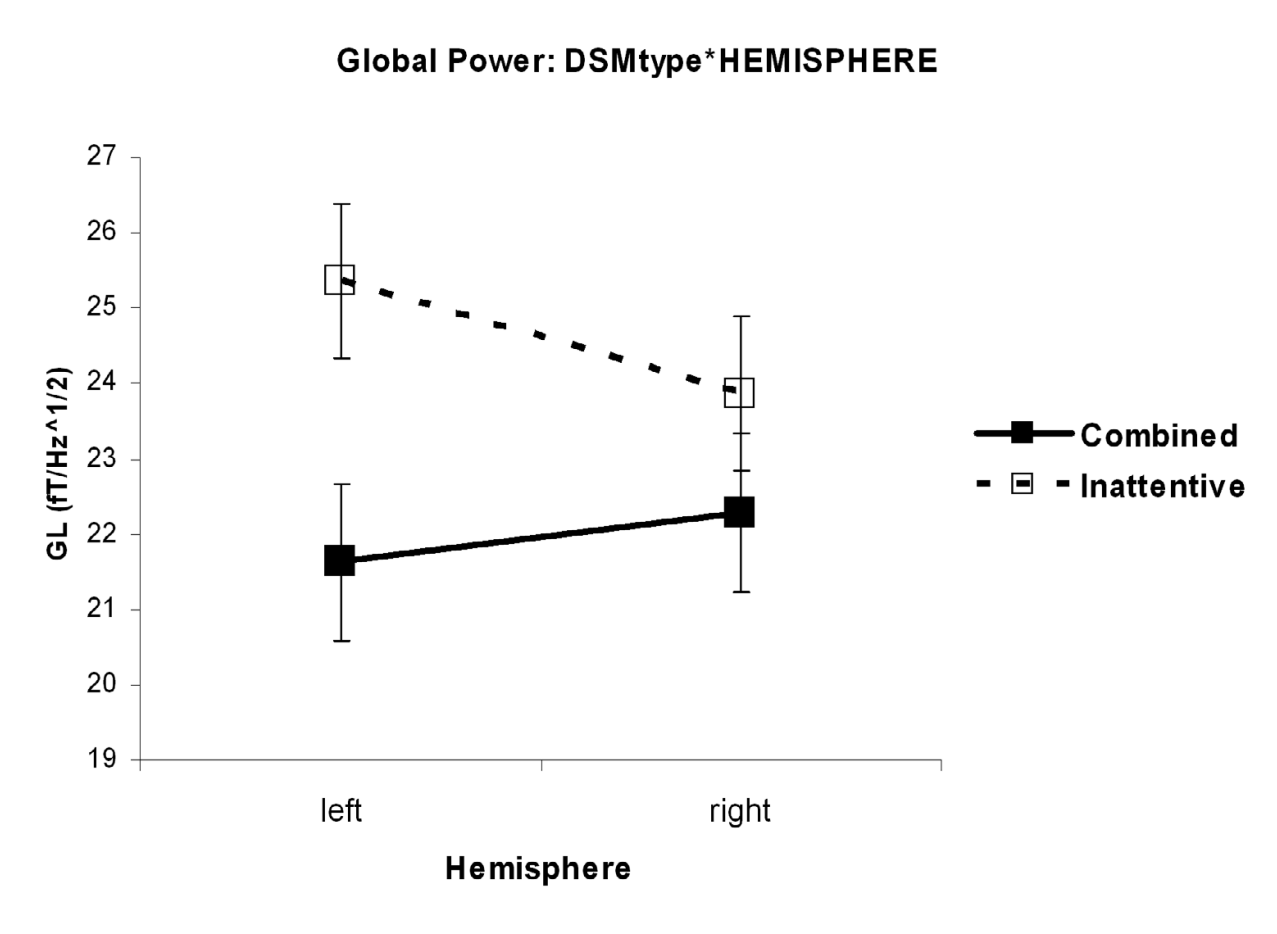
Interaction DSMtype*HEMISPHERE for GL. Independent of the administration of MPH ADHD children of the inattentive type showed a hemispheric asymmetry with higher global power amplitudes in the left hemisphere compared to the right hemisphere (p = 0.04). They also had higher global power amplitudes left hemispheric than ADHD children of the combined type (p = 0.07).

The interaction DSMtype*HEMISPHERE (F(1,33) = 7.79, p = 0.009) was revealed. Figure 5 displays that ADHDin children showed a hemispheric asymmetry with higher global power amplitudes in the left hemisphere compared to the right hemisphere (p = 0.04) independent of the administration of MPH. They also had higher global power amplitudes left hemispheric than ADHDcom children (p = 0.07).

#### Relative delta

The interaction DSMtype*HEMISPHERE (F(1,33) = 4.61, p = 0.039) was revealed. However, no differences were found in the post hoc analysis when p-values were adjusted following Tukey-Kramer. Looking at unadjusted p-values (p = 0.04), it was found that ADHDin children had lower relative delta band amplitudes in the right hemisphere (1.64) compared to the left hemisphere (1.66). No hemispheric differences were found for ADHDcom children.

#### Relative theta

**Figure 6:**
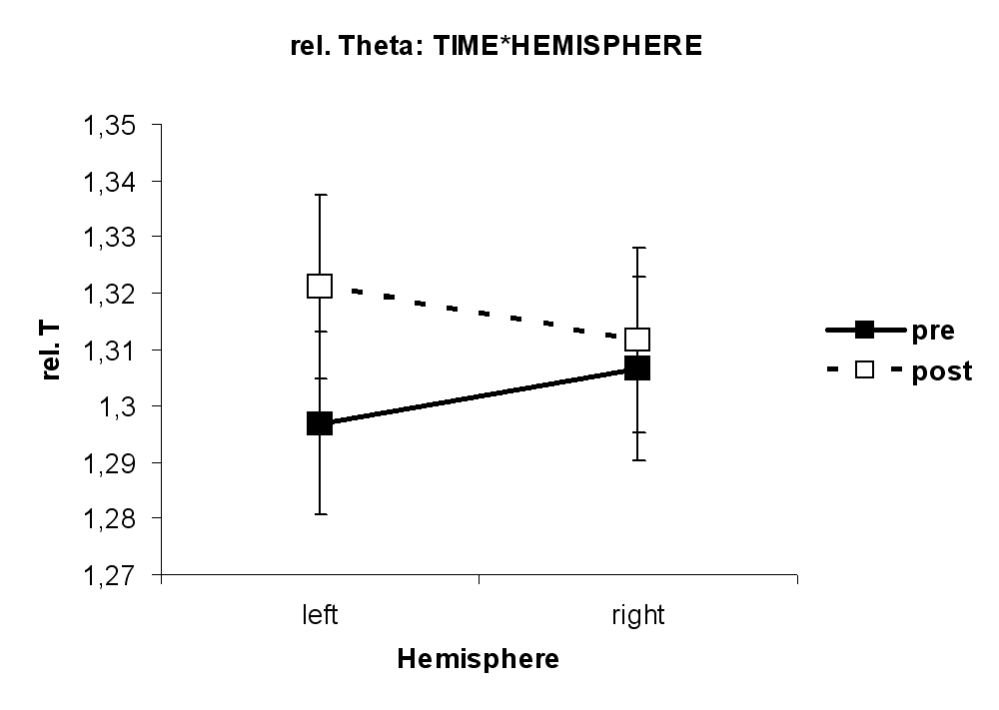
Interaction TIME*HEMISPHERE for relative Theta. In the left hemisphere relative Theta band amplitudes were higher after MPH than before MPH (p = 0.04).

**Figure 7:**
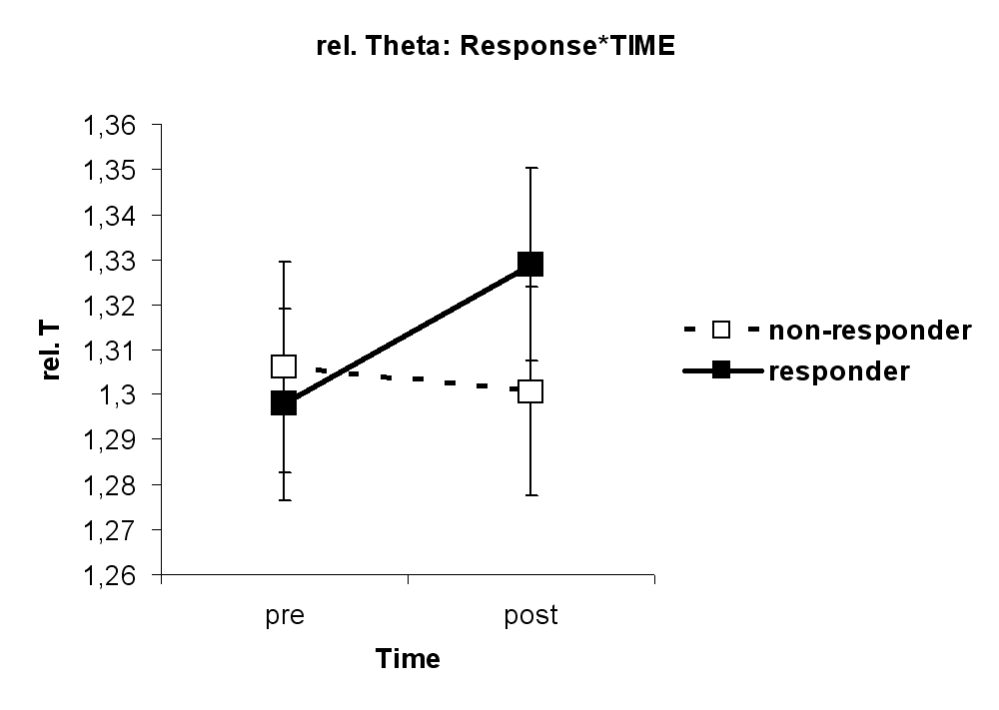
Interaction RESPONSE*TIME for relative Theta. ADHD children who responded to MPH had higher amplitudes in the relative Theta band after MPH than before MPH (p = 0.02).

The interaction TIME*HEMISPHERE (F(1,33) = 6.15, p = 0.018) was found. Figure 6 shows that in the left hemisphere relative Theta band amplitudes were higher after MPH than before MPH (p = 0.04). The interaction Response*TIME (F(1,33) = 6.16, p = 0.018) was revealed. As can be seen in figure 7, ADHDg children had higher amplitudes in the relative Theta band after MPH than before MPH (p = 0.02).

#### Relative alpha

**Figure 8:**
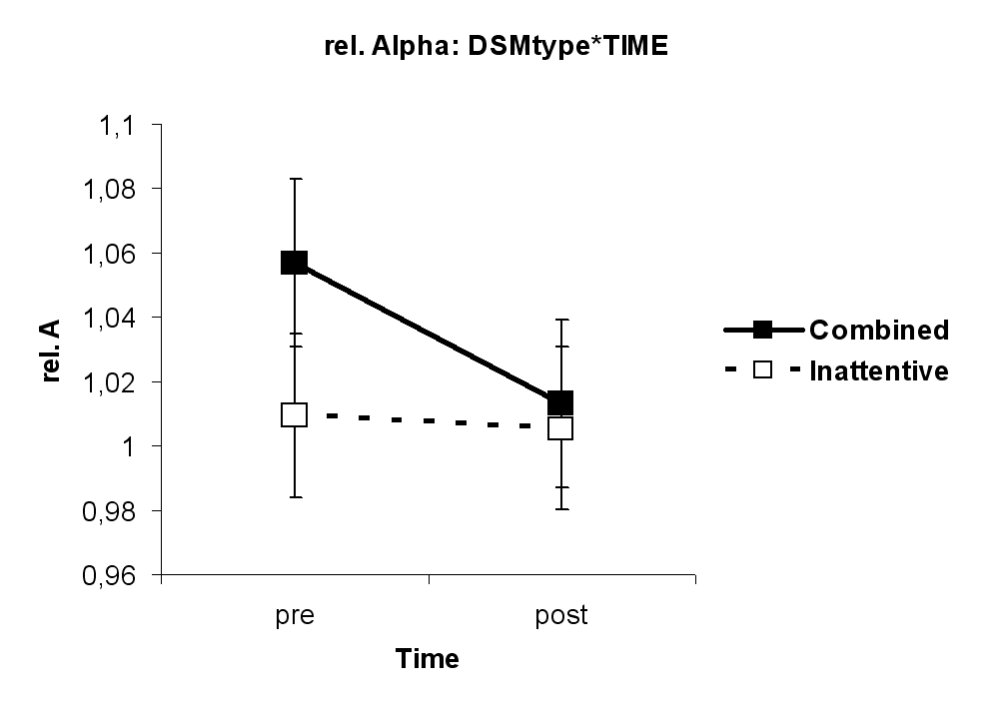
Interaction DSMtype*TIME for relative Alpha. ADHD children of the combined type had lower amplitudes in the relative Alpha band after MPH than before MPH (p = 0.009).

The main effect TIME (F(1,33) = 7.09, p = 0.012) was found. Amplitudes in the relative Alpha band were lower after MPH (1.01) than before MPH (1.03). The interaction DSMtype*TIME (F(1,33) = 4.9, p = 0.03) was revealed. ADHDcom children had lower amplitudes in the relative Alpha band after MPH than before MPH (p = 0.009, see fig. 8).

The main effect Response (F(1,33) = 6.27, p = 0.017) was found. ADHDg children (1.06) had higher relative Alpha amplitudes than ADHDp children (0.98).

#### Relative beta

The interaction TIME*HEMISPHERE (F(1,33) = 4.77, p = 0.036) was revealed. However, no differences were found in the post-hoc analysis.

The interaction Response*TIME (F(1,33) = 5.18, p = 0.029) was found. Again, effects did not prove to be significant in the post-hoc analysis.

#### Theta/alpha ratio

The main effect TIME (F(1,33) = 7.24, p = 0.01) was revealed. The Theta/Alpha ratio was higher after MPH (1.32) than before MPH (1.28).

The main effect Response (F(1,33) = 4.58, p = 0.0399) was revealed. The Theta/Alpha ratio was lower for ADHDg children (1.24) than for ADHDp children (1.37).

#### Theta/beta ratio

**Figure 9:**
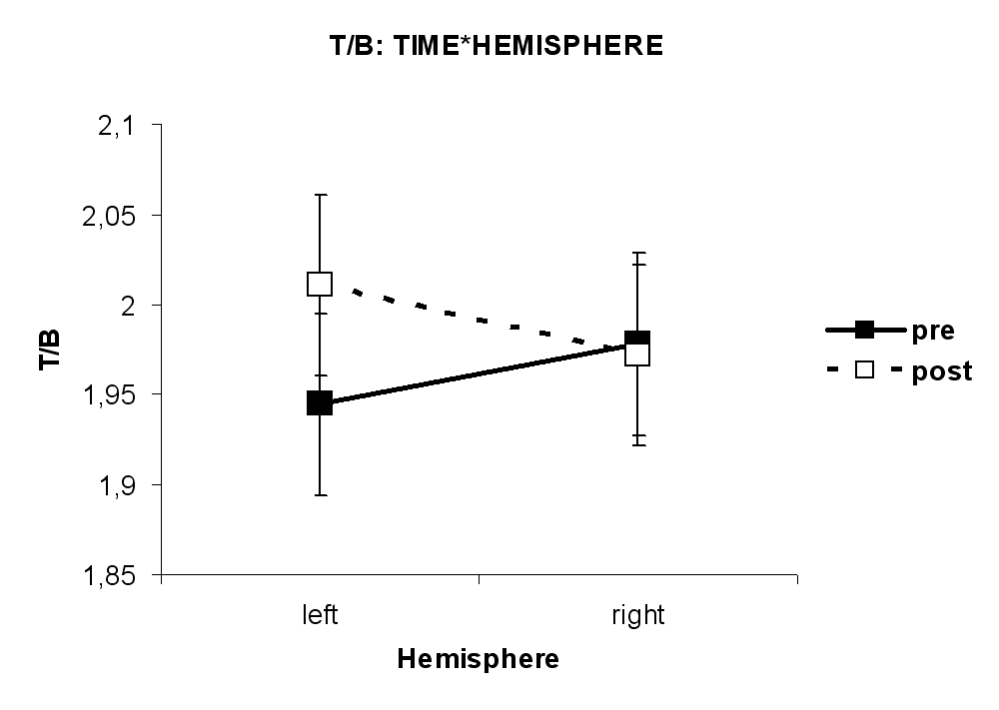
Interaction TIME*HEMISPHERE for T/B ratio. The Theta/Beta ratio was higher after administration of MPH than before MPH in the left hemisphere only (p = 0.02).

The interaction TIME*HEMISPHERE (F(1,33) = 6.74, p = 0.014) was found. However, no differences were found in the post hoc analysis when p-values were adjusted following Tukey-Kramer. Looking at unadjusted p-values, it was revealed that the Theta/Beta ratio was higher after MPH than before MPH in the left hemisphere (p = 0.02, see fig. 9).

**Figure 10:**
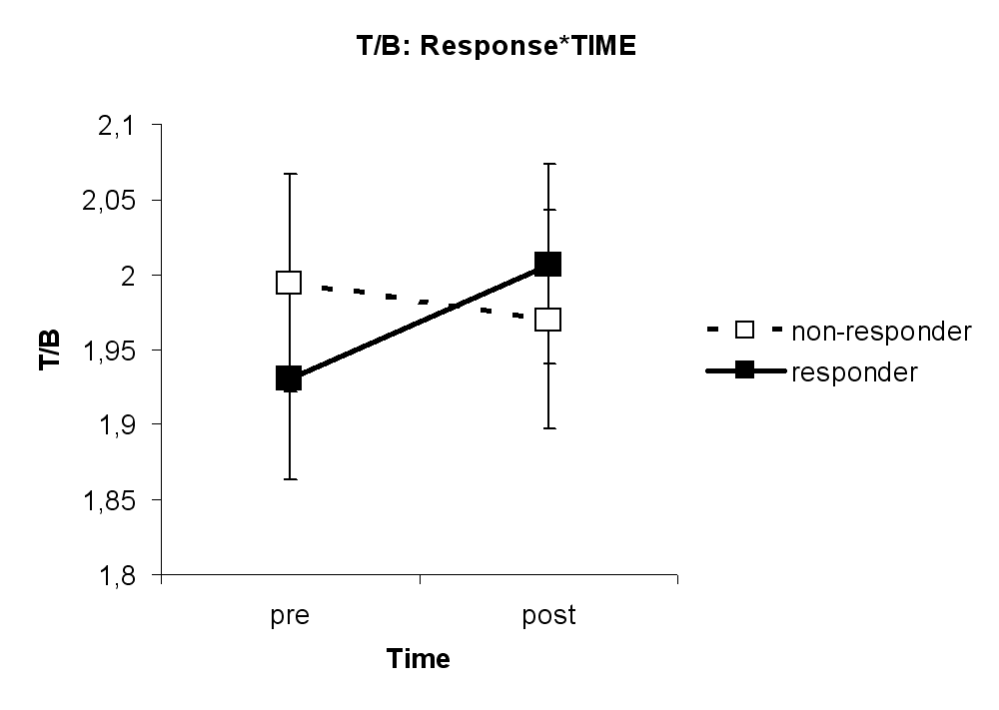
Interaction RESPONSE*TIME for T/B ratio. It was found that ADHD children who responded well to MPH had a higher Theta/Beta ratio after MPH than before MPH (p = 0.02).

The interaction Response*TIME (F(1,33) = 4.75, p = 0.037) was revealed. Again, no differences were found in the post hoc analysis when p-values were adjusted following Tukey-Kramer. Looking at unadjusted p-values, it was found that ADHDg children had a higher Theta/Beta ratio after MPH than before MPH (p = 0.02, see fig. 10).

### Correlations and linear regressions

**Figure 11:**
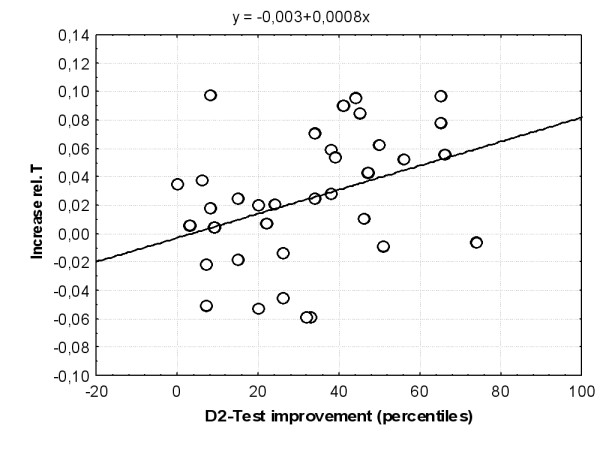
**Relationship between D2 test improvement and relative Thetapower increase. **A correlation was found between D2 test improvement and MPH-induced power changes in the relative Theta band left frontal (r = .37, p < .05). A linear regression was computed and confirmed that the larger the improvement in D2 test performance was, the larger was the increase in T after MPH application (t = 2.27, p = 0.03).

A correlation was found between D2 test improvement and MPH-induced power changes in the relative Theta band left frontal (r = .37, p < .05). A linear regression was computed and confirmed that the larger the improvement in D2 test performance was, the larger was the increase in Theta after MPH application (t = 2.27, p = 0.03), see fig. 11. No other correlations were found between any MPH-induced power band changes, MPH blood serum concentration and D2 test improvement.

## Discussion

In the present study, main effects induced by medication were found in frontal regions. This result is consistent with etiological hypotheses of ADHD, as well as the working mechanism of MPH. MPH-influence on frontal lobe acitivation in ADHD subjects was also found in a SPECT study by Lou and colleagues [11]. They found ADHD subjects having reduced bloodflow in frontal regions as well as enhanced bloodflow in motor areas. After application of MPH, this pattern normalized. Niedermeyer [7,8] interprets these findings as support of the "lazy frontal lobe" hypothesis underlying ADHD. He argues that the prefrontal cortex is not only involved in allocation and sustaining attention (e.g. [22,23]), but also in inhibiting motor activity (augmented motor activity being characteristic for ADHD). Langleben and colleagues [12] performed a SPECT study with ADHD children who were on and off MPH. When the subjects were not taking MPH, bloodflow was higher in the motor, premotor, and the anterior cingulate cortices. The authors concluded that brief discontinuation of MPH treatment is associated with increased motor and anterior cingulate cortical activity. Thus, it appears that if the prefrontal cortex is underactivated, both attentional processes and the inhibition of the motor cortex will be diminished.

MPH acts upon the prefrontal cortex via the neurotransmitter dopamine. Involvement of the dopaminergic system has been suggested in patients suffering from ADHD since the symptoms can be successfully treated with MPH, a potent blocker of the dopamine transporter (DAT) [24]. MPH is known to influence the dopaminergic system by blocking dopamine reuptake and in consequence enhancing the availability of dopamine in the synaptic gap [13]. Dopamine is densely distributed in the prefrontal cortex as well as the striatum and acts mainly on inhibitory neurons. By increasing the availability of dopamine MPH seems to enhance the inhibitory effect on motor activity.

Our aim was to find cortical differences between children who were diagnosed with different subtypes of ADHD as well as children who were good or poor responders to MPH. We were also interested in MPH-induced changes that were common to all ADHD children. First of all, global power (amplitude power of the combined frequency band from 1.5 to 25 Hz) increased with MPH. This may be taken as further evidence of the hypoarousal model of ADHD (e.g. [9,10]). The hypoarousal model assumes that ADHD results from cortical underarousal (compare "lazy frontal lobe" hypothesis). If MPH increases the amplitude of the global power band, one might hypothesize that it counteracts cortical underarousal. Other studies supporting the hypoarousal model found decreased bloodflow especially in prefrontal areas [11,25]. In the study performed by Lou and colleagues (see above), this underarousal could be remediated by MPH.

In the present study, Alpha activity decreased in both hemispheres with MPH. Alpha activity has been related to attentional processes (e.g. [26-28]). I.e. synchronized Alpha activity can be found in the EEG when subjects are relaxed and inattentive. Alpha activity lessens when attention is directed towards a stimulus [26,27,29,30]. Klimesch and colleagues [26] argue that during Alpha desynchronization, different neural populations start oscillating with different frequencies which in consequence leads to the disappearance of the dominant Alpha rhythm. Our results cannot be directly compared to the findings described above, since we did not investigate Alpha activity related to vigilance tasks. However, we found decreased Alpha power after MPH application. Knowing that MPH is used to treat ADHD symptoms like excess motor arousal and inattentiveness, it is not surprising to find decreased Alpha activity after MPH application given that Alpha activity relates to attentiveness. The Alpha effect in the present study is in line with the results of an EEG study by Loo and co-workers [31], who also reported decreased Alpha power after MPH. The authors link this effect to an increase in cortical arousal. A similar effect of decreased Alpha activity recorded over left fonto-central sites in the EEG after MPH was reported by Swartwood et al. [32]. In contrast to these findings, the same group found *increased *Alpha activity after MPH over the left frontal pole in the same study. However, the authors take this contradictory result as "difficult to interpret". Clarke et al. [33] reported increased Alpha activity after MPH application for children diagnosed with ADHD of the predominantly inattentive type. The authors take this as part of a normalization of the EEG, since unmedicated ADHD children have been reported to have lower levels of Alpha activation compared to controls. The contradictory findings concerning the effect of MPH on Alpha activity are difficult to explain. Yet, knowing from studies on attention (see above) that higher levels of attentiveness are related to a decrease in Alpha activity, an MPH-induced decrease in Alpha power seems more plausible than an Alpha increase.

In the present study, Theta band activity increased left hemispherically after MPH application. This finding contradicts the results of Clarke and co-workers [33], Swartwood and colleagues [32] and Loo et al. [31]. All of them found lower levels of Theta power after MPH application. Generally, higher slow wave activity has been reported in ADHD children compared to controls (e.g. [34,35]). This was interpreted as an indicator of maturational lag in brain functioning (e.g. [35-37]), since slow wave activity normally decreases from childhood to adulthood (e.g. [38]). In a study by Chabot and Serfontein [39] EEG measures of ADHD children were compared to a normative database. Their results disagreed with the maturational lag hypothesis, since the EEG profile of ADHD children did not resemble the EEG profile of children of any age. Another possibility to interpret increased slow wave activity is again offered by the hypoarousal model. Bresnahan and colleagues [40] hypothesized that increased slow wave activity in ADHD subjects might be an effect of decreased dopamine functioning which is in turn the origin of cortical underarousal. In line with this, Clarke and co-workers [33], as well as Loo et al. [31] interpret the MPH-induced decrease of Theta power with an increase in cortical arousal. Swartwood and colleagues [32] assume that MPH blocks slow-wave activity. Interestingly, Loo and colleagues [16] found differential MPH-induced effects on Theta-activity depending on the DAT1 risk allele status of the ADHD children. In an eyes-open resting condition, children who carried the DAT1 10R allele (considered the "risk" allele) showed a focal *increase *in left parietal Theta power. Children, who carried the DAT1 9R allele showed a decrease. Unfortunately, this effect was not discussed by the authors. It seems, however, that Theta activity cannot solely be related to drowsiness and hypoarousal, otherwise MPH should not increase its power as in Loo et al.'s or the present study, especially since Theta increase was positively correlated with D2-test improvement. Theta band activity has also been investigated in connection to working memory processes. For instance, "functional" Theta activity was found in an EEG study investigating visual word encoding [41,42]. Event-related Theta activity was largest for words that could later be recalled. The authors assume that theta synchronization is selectively related to the encoding of new information. Interestingly, Theta power was largest left hemispheric. This corresponds to our finding of a left hemispheric increase in Theta power after MPH application. Larson and colleagues [43] found that long-term potentiation in the hippocampus is optimal when the stimulation pattern mimics theta rhythm. But also Theta oscillations generated in frontal brain regions play an active role in memory maintenance [44]. Aftanas and colleagues [45] found a relation between Theta synchronization and an emotionally positive state and internalized attention. This effect was particularly prominent in left prefrontal regions. Gevins et al. [46] related the midline theta rhythm to intense concentration.

In conclusion, the results described above suggest that Theta activity does not necessarily mirror cortical underarousal. It can also reflect information processing, consolidation and attention. The subjects in our study did not have to perform any task. They only rested in the MEG with open eyes. Thus, it is unlikely that the Theta increase found corresponds to information encoding or memory processes. However, it is possible, that MPH increases the functional aspect of the Theta rhythm rather than increasing underarousal or drowsiness. Again, the increase in Theta power was correlated with an increase in behavioural performance in the attention test D2. Since we defined children to be MPH good responders or non-responders based on their increase in D2-test performance, it is not surprising that it was only the children who responded well to MPH who showed an increase in Theta power and Theta/Beta ratio.

In the present study, the Theta/Alpha ratio also increased with MPH application (mostly in the left hemisphere), as did the Theta/Beta ratio. The increase in the Theta/Beta ratio is of course a consequence of an increase in relative Theta and a concurrent decrease in relative Alpha power. Thus, it does not reveal any new information. Presumably, the same is true for the increase in the Theta/Beta ratio. Although a significant interaction was found between Beta Power and MPH status, no significant differences were found in the post hoc tests. This implies that Beta power did not change to a great degree with MPH application and the increase in the Theta/Beta ratio is very likely a result of an increase in Theta power alone.

We did find differences between the two ADHD subtypes. Children with ADHD of the predominantly inattentive type had higher global power amplitudes in the left hemisphere than children with ADHD of the combined type. If global power activity reflects cortical arousal in our study (see above), one might hypothesize, that children with ADHD of the combined type are more hypoaroused than children with ADHD of the predominantly inattentive type. Clarke and colleagues [19] found the opposite result (combined > inattentive). They stated that higher levels of global power amplitude reflect cortical underarousal and consequently concluded that children with ADHD of the predominatly inattentive type are less hypoaroused than children with ADHD of the combined type. Yet, as described above, we found an MPH-induced increase in global power activity that was accompanied by an increase in behavioural performance. Thus, in our study, higher global power does not seem to mirror cortical hypoarousal, but in fact the opposite. Therefore, we might also conclude from our data that children with ADHD of the predominantly inattentive type are the ones being less hypoaroused. Another characteristic of children with ADHD of the inattentive type was a hemispheric asymmetry in global power and relative delta activity with more power in the left hemisphere. No asymmetries were found for children with ADHD of the combined type. Characteristic for the latter group was an MPH-induced decrease in Alpha power. Although the decrease in Alpha power became statistically significant for all ADHD children (see above), the effect seemed mainly to be driven by the children with ADHD of the combined type.

## Competing interests

The author(s) declare that they have no competing interests.

## Authors' contributions

CW designed the study, carried out the data acquisition, data processing and statistics, drafted the manuscript and has given final approval of the version to be published.

IP made substantial contribution to study design, carried out data acquisition and statistics and drafted the manuscript.

SB carried out the subject selection, performed the neuropsychological testing, and contributed during data acquisition.

HK carried out subject diagnostics, medical treatment, and contributed to data acquisition and the preparation of the manuscript.

## Pre-publication history

The pre-publication history for this paper can be accessed here:


